# *Festuca drakensbergensis* (Poaceae): A common new species in the *F. caprina* complex from the Drakensberg Mountain Centre of Floristic Endemism, southern Africa, with key and notes on taxa in the complex including the overlooked *F. exaristata*

**DOI:** 10.3897/phytokeys.162.55550

**Published:** 2020-10-07

**Authors:** Steven P. Sylvester, Robert J. Soreng, Mitsy D.P.V. Sylvester, Vincent Ralph Clark

**Affiliations:** 1 College of Biology and the Environment, Nanjing Forestry University, Long Pan Road No. 159, Nanjing, 210037, China Nanjing Forestry University Nanjing China; 2 Department of Botany, National Museum of Natural History, Smithsonian Institution, Washington DC 20013-7012, USA Smithsonian Institution Washington United States of America; 3 Afromontane Research Unit and Department of Geography, University of the Free State, Qwaqwa Campus, Phuthaditjhaba, 9866, South Africa University of the Free State Phuthaditjhaba South Africa

**Keywords:** alpine grassland, Gramineae, *Festuca caprina*, Flora of Southern Africa, Lesotho, Maloti-Drakensberg, South Africa

## Abstract

We present taxonomic notes on the *Festuca caprina* complex from southern Africa that includes description and illustration of the new species *F. drakensbergensis* from the Drakensberg Mountain Centre of Floristic Endemism of South Africa and Lesotho. *Festuca drakensbergensis* can be differentiated from *F. caprina* s.l. by forming lax short tufts with extravaginally-branching tillers and lateral-tending cataphyllous shoots or rhizomes present, basal foliage reaching < ½ the length of the culms, with generally shorter leaves and shorter anthers, 0.8−1.6(−1.8) mm long. The species also differs from the overlooked species *F. exaristata* – currently known from two collections from Lesotho − by its fibrous basal sheaths, usually sharp, keel-like leaf blade midrib, drooping panicle with lightly to densely scabrous pendent panicle branches, longer lemmas, 4.5−5.8 mm long, with awns usually present, 0.5–3 mm long, ovary apices sparsely to densely hairy and anthers 0.8−1.6(−1.8) mm long. Taxonomic notes on the different taxa of the *F. caprina* complex in southern Africa are also provided, including images, key, and lectotypification of F. caprina var. curvula. This research adds a further two endemic species (*F. drakensbergensis* and *F. exaristata*) and two endemic varieties (F. caprina var. irrasa and F. caprina var. macra) to the Drakensberg Mountain Centre of Floristic Endemism.

## Introduction

Carbutt’s (2019) Drakensberg Mountain Centre of Floristic Diversity and Endemism (DMC) includes the only alpine region in mainland Africa south of Mount Kilimanjaro (Killick 1978), with a 2900 km disjunction. The DMC, covering some 40,000 km^2^, comprises a montane sub-centre, dominated by C_4_ grass species and an alpine sub-centre [the former Drakensberg Alpine Centre of van Wyk and Smith (2001) and Carbutt and Edwards (2004, 2006)] dominated by C_3_ grass species (Brand et al. 2019). The DMC is renowned for its high levels of plant diversity and endemism, hosting 227 endemic angiosperm species that account for ca. 9% of the angiosperm flora; the DMC hosts 267 grass species in 86 genera (Carbutt and Edwards 2004), of which eight species and one genus are endemic (Carbutt 2019). Despite being the dominant ecosystem-forming component of these high elevation grasslands, grasses of the DMC are still relatively poorly studied, with only a few genera receiving attention, for example, *Anthoxanthum* L. (Mashau 2016); *Catabrosa* P. Beauv. (Soreng and Fish 2011); *Poa* L. (Soreng et al. in prep.); *Trisetopsis* Röser & A. Wölk (e.g. Mashau et al. 2010); *Pentameris* P. Beauv. (Linder and Ellis 1990).

The genus *Festuca* L. s.l. is a monophyletic lineage with ca. 650 perennials and ca. 30 annuals (beyond those in *Lolium* L.), totalling ca. 680 species (Plants of the World Online 2020 accepted species belonging to the lineage). The genus s.l. is divided into two major clades (Minaya et al. 2017): the Narrow Leaf Clade (NLC) of *Festuca* s.s., ca. 600 species (syn. [following Soreng et al. 2017 including the annuals] *Ctenopsis* De Not., *Loliolum* V.I. Krecz. & Bobrov, *Micropyrum* (Gaudin) Link, *Narduroides* Rouy, *Vulpia* C.C.Gmel. and *Wangenheimia* Moench), and the Broad Leaf Clade (BLC), ca. 82 species (perennials, and some annuals in *Lolium*), including *Drymochloa* Holub, *Leucopoa* Griseb., *Lojaconoa* Gand., *Lolium* (syn. *Micropyropsis* Romero Zarco & Cabezudo, *Schedonorus* P. Beauv.), *Patzkea* G.H. Loos, *Pseudobromus* K. Schum. and *Xanthochloa* (Krivot.) Tzvelev.

Fish and Moeaha (2015) accepted nine species of *Festuca* s.l. (but excluding *Vulpia* and *Lolium* in the narrow traditional sense) as present in the Flora of Southern Africa (FSA) region (comprising Botswana, Lesotho, Namibia, South Africa and Eswatini a.k.a. Swaziland). Generic limits of *Festuca* s.l. are still being resolved, particularly in the BLC (Soreng et al. 2017). Of the FSA species with DNA examined (Minaya et al. 2017), *F. caprina* Nees and *F. vulpioides* Steud. belong to the NLC, whereas *F. arundinacea* Schreb. (= *Lolium arundinaceum* (Schreb.) Darbysh.), *F. africana* (Hack.) Clayton (= *Pseudobromus silvaticus* K. Schum.), *F. costata* Nees, *F. killickii* Kenn.-O’Byrne, *F. longipes* Stapf and *F. scabra* Vahl belong to the BLC. Although it generally holds true, not all NLC and BLC taxa have narrow and broad leaves, respectively, for example, *F. vulpioides* being placed in the NLC (Minaya et al. 2017; identity of voucher specimen not verified by us). *Festuca dracomontana* H.P. Linder (predicted to be BLC), *F. exaristata* E.B. Alexeev (not accounted for by Fish and Moeaha 2015, predicted to be NLC) and our new species (predicted to be NLC) have not been tested.

*Festuca* s.l. is one of the prominent genera present in the montane-alpine ecotone (ca. 2500–2800 m alt.) and alpine sub-centre (> 2800 m alt.) of the DMC (Irwin and Irwin 1992) and often dominates, especially in less disturbed areas (Sylvester et al. unpubl. data). One species, *F. killickii*, is currently considered to be endemic to the DMC (Carbutt 2019: table 2), although the poorly-known *F. dracomontana* and *F. vulpioides* may also be DMC endemics (Fish and Moeaha 2015). Of the species of *Festuca* recorded by Fish and Moeaha (2015), *F. caprina* is perhaps the most widespread in the Afro-montane/Afro-alpine region of White (1981), stretching from the coastal Southern Cape of South Africa to Tanzania (Fish and Moeaha 2015). *Festuca caprina* s.l. has had three varieties described from the FSA region (var. curvula Nees, var. irrasa Stapf, var. macra Stapf) and was considered to be a complex of species by Alexeev (1986), who recognised two new species for the complex in sub-Saharan Africa, *F. claytonii* E.B. Alexeev from Kenya and *F. exaristata* E.B. Alexeev from the DMC, and raised F. caprina var. macra to species rank. Fish and Moeaha (2015: 349) stated that the different varieties of *F. caprina* accepted in previous treatments were not upheld in their treatment because of “the variability in the species and leaf anatomy, which are constant throughout”. Although Alexeev’s (1986) taxonomy and new species were accepted by agrostologists at Kew (Phillips 1995a, b; Clayton et al. 2006 onwards), there is no mention of it in Fish and Moeaha (2015) or the older treatment of *Festuca* for the FSA region (Gibbs Russell et al. 1990) and the checklist of Lesotho grasses (Kobisi and Kose 2003), with this error also being replicated in floristic surveys of the DMC (Carbutt and Edwards 2004, 2006; Carbutt 2019).

Taxa in the *F. caprina* complex differ from other *Festuca* s.l. taxa in the FSA region by having: basal sheaths entire or splitting into narrow parallel threads (vs. coarsely fibrous in *F. costata*), glabrous or scabrous (vs. basal ones velvety in *F. scabra*); ligules < 1 mm long (vs. > 1 mm long in most, apart from *F. dracomontana* and *F. vulpioides*); collars non-auriculate (vs. auriculate in *F. arundinacea*, *F. dracomontana* and *F. vulpioides*); blades narrow, 0.2–1.5 mm wide in diameter, involute (vs. flat or relatively broad, [2–]3–15 mm wide in diameter, rarely narrower in *F. scabra*); panicles loose or contracted (vs. very open, candelabrum-shaped, in *F. longipes*, open in *F. africana*, *F. arundinacea* and *F. dracomontana*); spikelets 2 to several flowered (vs. 1-flowered in *F. africana*), awns 0–5.5 mm long (vs. 10–20 mm long in *F. africana*).

During extensive field collecting and ecological research by the authors in the DMC area (222 2 m × 2 m plots studied for all vascular plants, of which 145 plots contained *Festuca* species, with 50 collections of *Festuca* made), followed by herbarium research at PRE, clear differences were noted between specimens that were treated under *F. caprina* by Fish and Moeaha (2015). These differences included branching patterns in tillers, presence of cataphylls, abaxial leaf blade indumentum and anther size, which are known to be taxonomically informative for distinguishing *Festuca* taxa in other parts of the World (e.g. Stančik and Peterson 2007; Ospina et al. 2015). These clear differences allowed us to distinguish the new species, *F. drakensbergensis*, and to recognise the varieties F. caprina var. irrasa and F. caprina var. macra. This new species, coupled with the overlooked species, *F. exaristata* and distinct varieties, F. caprina var. irrasa and var. macra, add a further two endemic species and two endemic varieties to Carbutt’s (2019) checklist of DMC endemics.

The aim of this paper is therefore to:

(i) Describe and illustrate the new DMC endemic, *F. drakensbergensis*.

(ii) Provide taxonomic notes on the distinct varieties of *F. caprina* present in the DMC and the overlooked species, *F. exaristata*.

(iii) Provide a revised key for the *F. caprina* complex in the FSA region.

## Materials and methods

Extensive field collecting was conducted by SPS, RJS and MDPVS in the DMC between 1 Feb and 9 Mar 2020, with 42 specimens belonging to the *F. caprina* complex collected, which are deposited in the PRE, NU and US (pending export permits) herbaria [Herbarium acronyms follow Thiers (2020, continuously updated)]. Herbarium study was also conducted at PRE between 13 and 20 Mar 2020. While focus was placed on the 42 new field collections of *Festuca* and notes on variations present in our 142 plots containing taxa belonging to the *F. caprina* complex, many other older PRE herbarium specimens were studied than mentioned in the ‘Selected specimens examined’ sections herein, but, due to unforeseen obstructions caused by the COVID-19 pandemic, information regarding these specimens was not adequately recorded. Type images on JSTOR Global Plants (https://plants.jstor.org) were also assessed. We delimit taxa based on distinct discontinuities in morphological characteristics which are deemed to be phylogenetically conserved and taxonomically informative based on previous research (e.g. Stančik and Peterson 2007; Ospina et al. 2015), as well as distinct discontinuities in ecological and morphological characteristics of taxa observed during extensive fieldwork in the DMC area. Distinctive characteristics of habit, colouration and ecological preferences, notable between individual plants within and amongst populations in the field, are often difficult to sort out when dealing only with herbarium specimens. In this treatment, glabrous means without pubescence (in the sense of slender, relatively soft hairs, unless otherwise stated). Smooth indicates no prickle-hairs with broad bases and/or hooked or pointed apices (i.e. pubescence can occur on a smooth surface and a rough or scabrous surface can be glabrous). Leaf-blade anatomical characteristics were observed in cross-sections from the middle area of selected tiller blades. We collected many silica-dried leaf samples of *Festuca* s.l. for future DNA examinations.

## Taxonomic treatment

### Key to species of the *Festuca caprina* complex in southern Africa

Key characters separating species of the *F. caprina* complex in southern Africa are also found in Table 1.

**Table:** 

1	Tillers intravaginal (cataphylls absent, elongated prophylls present at juncture of lateral shoots), lateral tending rhizomes absent; densely tufted and usually forming large tussocks with basal foliage reaching (10−)20–80+ cm tall and often > ½ the length of the culms; sheaths of tillers and basal culm (3–)12–24 cm long; leaf blades of tillers and basal culm (4−)12−66.5+ cm long; lowermost lemmas (4.5−)5−7(−9) mm long; fertile anthers (1.8−)2−4 mm long (as short as 1.6 mm in var. macra, according to Alexeev 1986) (*F. caprina* s.l.)	**2**
–	Tillers extravaginal (rarely some intravaginal shoots also present), lateral-tending or ascending cataphyllous shoots or lateral-tending rhizomes present; plants forming lax short tufts with basal foliage reaching (2−)4−20(−27) cm tall and < ½ the length of the culms; sheaths of tillers and basal culm (0.5–)2–7(–10) cm long; leaf blades of tillers and basal culm (2–)5–15(–26) cm long; lowermost lemmas 4−5.8 mm long; fertile anthers 0.8−1.8 mm long	**4**
2	Sheaths of old leaves falling apart (shredded) into parallel thin threads; basal foliage ca. 14–30 cm tall, often < ½ the length of the culms; panicle branches and pedicels short-hispid or long-scabrous with hair-like prickles; lemmas, paleas and rachillas short-hispid or long-scabrous with hair-like prickles; lemma apices usually notably bifid, with awn emerging from between the lobes	**F. caprina var. irrasa Stapf**
–	Sheaths of old leaves entire, not or rarely only very slightly disintegrating into fibres; basal foliage (10−)30–80+ cm tall, generally (> ½) > ¾ to surpassing the length of the culms; panicle branches and pedicels short scabrous; lemmas, paleas and rachillas glabrous, scabrous, but prickles hooked, short hooked, slender or stout, not hair-like, rarely smooth; lemma apices not usually notably bifid, commonly merging into the awn	**3**
3	Leaf blade abaxial surface antrorsely scabrous throughout	**F. caprina var. macra Stapf**
–	Leaf blade abaxial surface smooth or rarely antrorsely scaberulous towards the apex	**F. caprina var. caprina Nees**
4	Sheaths of old leaves falling apart (shredded) into parallel thin threads; leaf blade midrib (middle vein) usually sharp, keel-like, sometimes blunt and rounded; panicles drooping; panicle branches usually pendant, lightly to densely scabrous; lowermost lemma (not including awn) 4.5−5.8 mm long; awn usually present, very rarely muticous, awn 0.5–3 mm long; ovary apex sparsely to densely hairy; fertile anthers 0.8−1.6(−1.8) mm long; basal foliage reaching (2−)4−20(−27) cm tall	***F. drakensbergensis* Sylvester, Soreng & M.D.P.V. Sylvester**
–	Sheaths of old leaves entire, not disintegrating into fibres, lustrous; leaf blade midrib (middle vein) blunt, rounded; panicles erect; panicle branches smooth; lowermost lemma (not including awn) 4−4.2 mm long; awn absent; ovary apex glabrous; fertile anthers 1.5−1.8 mm long; basal foliage reaching to 12 cm tall	***F. exaristata* E.B. Alexeev**

**Table 1.** Differences in key morphological characters between the species of the *Festuca caprina* complex in southern Africa.

Character	F. caprina var. caprina	F. caprina var. irrasa	F. caprina var. macra	*F. exaristata*	*F. drakensbergensis*
Tillers	intravaginal			Extravaginal, rarely also intravaginal	
Culm height (cm)	35–100	ca. 30–65	(28–)60–110(–120+)	ca. 19–35	(12.5–)20–46(–65)
Height of basal foliage	(20−)30–60+ cm tall, generally (> ½) > ¾ to surpassing the length of the culms	ca. 14–30 cm tall, often < ½ the length of the culms	(10−)30–80+ cm tall, generally > ½ the length of the culms	ca. 12–25 cm tall, generally < ½ the length of the culms	(2−)4−20(−27) cm tall, generally < ½ the length of the culms
Sheaths of old leaves	Not falling apart into parallel thin threads	falling apart (shredded) into parallel thin threads	Not falling apart into parallel thin threads		Falling apart (shredded) into parallel thin threads
Sheaths of tillers and basal culm length (cm)	ca. 6–16(–24)	ca. 2–10	(3–)12–24+	ca. 1–4(–10?)	(0.5–)2–7(–10)
Basal culm and tiller leaf blade length (cm)	(8.5–)12–60	ca. 1.8–20	(4–)13–66.5+	ca. 2–25	(2–)5–15(–26)
Leaf blade in middle vein (midrib)	Sharp, keel-like			Blunt, rounded	Usually sharp, keel-like, sometimes blunt, rounded
Abaxial leaf surface	Smooth or rarely scaberulous towards apices	Smooth or rarely scaberulous towards apices	Scabrous throughout	Smooth	Smooth or only scaberulous at apex
Panicle branches	Scabrous	Usually long-scabrous, prickles hair-like	Scabrous	Smooth	Scabrous or rarely smooth
Lowermost lemma length (mm)	(4.5−)6−7(−9)	ca. (5.2−)5.5−6.5	(4.5−)5−7.2(−9?)	4−4.2	4.5−5.8
Spikelet pubescence	glabrous	Usually hispid on lemmas, paleas and rachillas	glabrous		
Awn length (mm)	(0−)1−4.5	ca. 1.5−2.8	(0−)1.5−5.5	0	(0−)0.5−3
Anthers length (mm)	(2.1−)2.4−4	ca. 2.6−2.8	(1.6 mm?; Alexeev 1986)(1.8−)2−3.5(−4)	1.5−1.8	0.8−1.6(−1.8)
Ovary apex	Hairy, hairs sparse (sometimes just 1 or 2) or dense			Glabrous	Hairy, hairs sparse or dense

#### Festuca drakensbergensis

Taxon classificationPlantaePoalesPoaceae

Sylvester, Soreng & M.D.P.V. Sylvester sp. nov.

502C9104-6F90-5210-AEF8-C13618534904

urn:lsid:ipni.org:names:77211930-1

**Figure 1.**
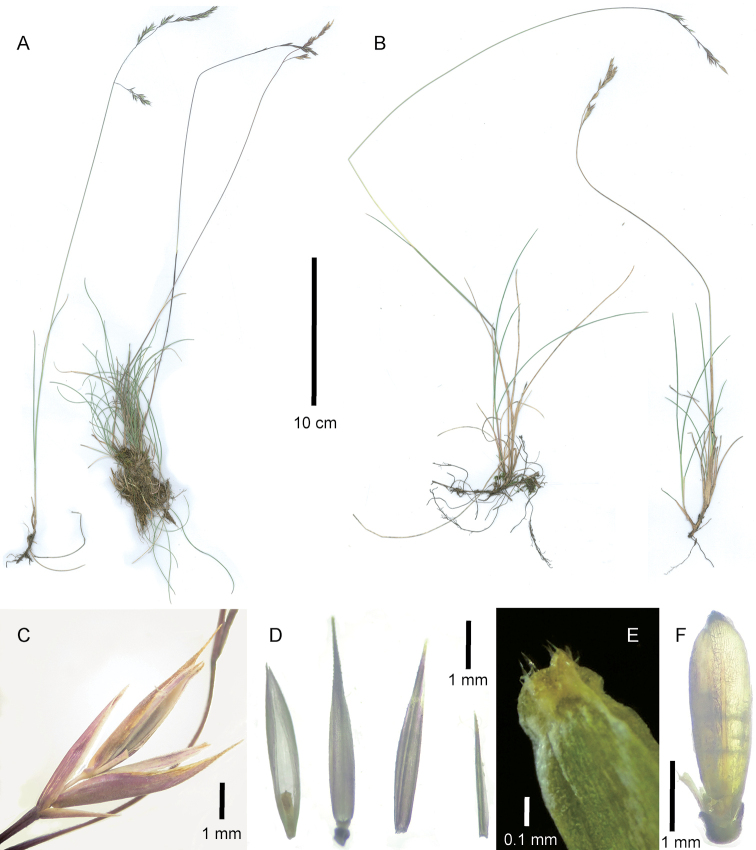
*Festuca drakensbergensis*, habit and inflorescence characteristics. **A, B** Whole plant **C** spikelet, lateral view **D** [from left to right] palea ventral view showing ovary, lemma dorsal view, upper glume dorsal view, lower glume lateral view **E** ovary apex, ventral view **F** caryopsis, dorsal view, with parts of torn palea and lemma at base. **A, C, D, F** of isotype S.P. Sylvester et al. 3660 (US) **B** of S.P. Sylvester et al. 3578 (US) **E** of S.P. Sylvester et al. 3687a (PRE).

**Figure 2.**
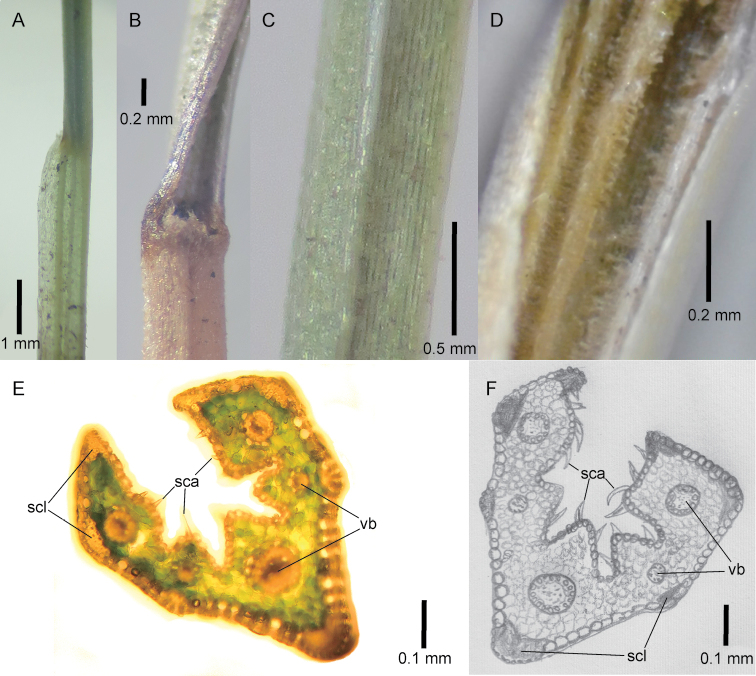
*Festuca drakensbergensis*, leaf morphological and anatomical characteristics. **A** Junction of tiller sheath and blade, lateral view **B** ligule of tiller, ventral view **C** abaxial tiller blade surface, showing keel **D** adaxial tiller blade surface **E, F** tiller blade cross sections, showing position of the sclerenchyma block (scl), vascular bundles (vb) and scabers (sca) on the adaxial surface. **A, B, C, E** of isotype S.P. Sylvester et al. 3660 (US) **D** of S.P. Sylvester et al. 3689 (PRE) **F** of isotype S.P. Sylvester et al. 3660 (US) drawn by M.D.P.V. Sylvester.

Figs 1 , 2 , Table 1

##### Type.

Lesotho. AfriSki Ski Resort, in valley just west of the resort centre with an east-southeast aspect, 28.824908S, 28.723208E, 3065 m alt., heavily grazed damp Afro-alpine grassland, 28 Feb 2020, S.P. Sylvester, R.J, Soreng & M.D.P.V. Sylvester 3660 (holotype: PRE!; isotypes: NU!, US!).

##### Diagnosis.

Differs from *Festuca caprina* s.l. by forming lax short tufts with extravaginally branching tillers and lateral-tending or ascending cataphyllous shoots or lateral-tending rhizomes present, basal foliage reaching < ½ the length of the culms, sheaths of tillers and basal culm (0.5–)2–7(–10) cm long, leaf blades of tillers and basal culm (2–)5–15(–26) cm long, and anthers 0.8−1.6(−1.8) mm long. Differs from *Festuca exaristata* by its basal sheaths fibrous, leaf blade midrib usually sharp, keel-like, sometimes blunt and rounded, panicle branches pendent, lightly to densely scabrous, lowermost lemma (not including awn) 4.5−5.8 mm long, awn usually present, 0.5–3 mm long, ovary apex sparsely to densely hairy and anthers 0.8−1.6(−1.8) mm long.

##### Description.

Perennial herbs, generally forming lax, short, isolated tufts, with lateral-tending or ascending cataphyllous shoots or lateral-tending rhizomes present, basal foliage (2−)4−20(−27) cm tall and generally < ½ the length of the culms, with inflorescences largely exerted. ***Tillers*** extravaginal, with cataphylls present, intravaginal tillers rarely also present (i.e. Sylvester et al. 3637). ***Culms*** (12.5–)20–46(–65) cm tall, 0.3–0.5(–1) mm diam., erect, delicate, cylindrical to slightly compressed, longitudinally striated, glabrous, smooth, with (0) 1 or 2 visible nodes, uppermost node at (1.3–) 3–10(–16) cm from the base, ca. (1/10–)1/8–1/3(–½) culm height, distance between uppermost node and panicle (3–)14–33(–40) cm long, distance between uppermost node and second node down (0.9–)2.3–6.5(–9.5) cm long, nodes at the base covered by imbricate leaf sheaths. ***Leaves*** mostly basal, with 1 or 2 (3) cauline leaves, culm leaves similar to those of the base and tillers; ***sheaths*** of tillers and basal culm (0.5–)2–7(–10) cm long, proximally fused ca. ½ their length, implicate above, usually slightly obliquely truncated at the apex, herbaceous, persistent, becoming sparingly fine fibrous – decaying into longitudinal fibres – in the lower portion with age, brownish or yellowish, glabrous, usually smooth, rarely retrorsely scabrous, with 5–7 veins; flag-leaf sheaths 3.4–9.5(–12.5) cm long, fused ca. ½ their length; ***auricles*** 0.01–0.2 mm long, inconspicuous, obtuse; ***ligules*** 0.1–0.5 mm long, membranous, moderately to strongly decurrent with the sheath margins, truncate, briefly ciliolate; flag-leaf ligules 0.2–0.5 mm long; ***leaf blades*** of tillers and basal culm (2–)5–15(–26) cm long, 0.3–0.8(–1) mm wide as rolled or folded, setaceous, erect-curved to recurved, firm to ± rigid, conduplicate, convolute or involute, rarely flat in upper leaves, elliptical or obovate to carinate outline in cross-section, midrib (middle vein) usually sharp, keel-like, sometimes blunt and rounded, abaxial surface glabrous, usually smooth throughout or lightly antrorse-scabridulous towards the apex, adaxial surface scabrous on veins or prickles elongating to become hair-like and appearing shortly hairy, light- to dark-green, apex obtuse (to acute); upper culm leaf-blades similar to those of lower culm and tillers, but shorter and sometimes expanded; flag-leaf blades (0.2–)1.5–4(–12.5) cm long, (2–)15–40(–50)% the length of their flag-leaf sheaths, rarely longer. ***Panicles*** 2.5–9(–13) cm long, open to moderately congested, drooping, with (7–)8–20(–50) spikelets often held unilaterally on lower side of axis; central panicle axis smooth to lightly antrorsely scabrid, with 4–10 nodes, usually 1 branch (rarely 2 branches) per node, lowest internode (0.8–)2–4.5(–5.5) cm long, ca. 20–70% length of whole panicle, lowest internode and sometimes upper internodes and panicle branches often sinuous-wavy; ***panicle branches*** capillaceous, generally pendent and drooping, lowermost patent to pendent, upper ± appressed to central axis, glabrous, antrorsely scabrous to scaberulous on angles or rarely smooth; lowermost primary panicle branch (1–)1.5–6 cm long, with (1–)3–10(–17) spikelets; ***pedicels*** 0.5–3(–6) mm long, shorter than their spikelets, slightly thickened at their apices, glabrous, antrorsely scabrous to scaberulous on angles or rarely smooth. ***Spikelets*** (not including awns) (5.5–)6–9(–11.5) mm long, laterally compressed, elliptic, green or usually purplish; ***florets*** 2 to 5(6) fertile and usually 1 apical and ± rudimentary, sterile, lowermost fertile floret largest, with upper fertile florets gradually reducing in size; ***glumes*** unequal, lower ca. ½–3/4(–5/6) length and ca. 1/3–½ width of upper glume, narrowly scarious on the margins, usually darker purple compared to the lemmas, glabrous, keels distally scaberulous for ¼–1/2 their length or smooth throughout, surfaces smooth throughout or sometimes sparsely scaberulous towards apex, margins usually with scattered hooks on edges in distal ½(–3/4), (acute or) acuminate; ***lower glumes*** 2.1–3(–3.8) mm long, 0.3–0.4 mm wide at base in cross section, reaching to 50–70% length of proximal lemma, linear-lanceolate, 1-veined; ***upper glumes*** 3.2–4(–4.9) mm long, 0.5–0.8 mm wide at base in cross section, reaching to 70–95% length of proximal lemma, ovate-lanceolate, 3-veined; ***rachillas*** up to ca. 0.8–1.6 mm long, slightly dorsally compressed, glabrous, smooth, lightly scabrous towards apex or densely scabrous throughout; ***calluses*** somewhat thick, annulated, angled downward, rugose or smooth, sometimes lightly scabrous; ***lemmas*** (lowermost lemma not including awn) 4.5–5.8 mm long, 0.7–1.2 mm wide at broadest point in cross section, ovate-lanceolate, herbaceous with narrowly scarious margins, glabrous, proximally smooth or sparsely to densely scabrous, especially towards the margins, distally sparsely to densely scabrous, especially towards the apex and margins, moderately to densely granulose with clear bead-like raised silica cells appearing like ‘granules’ throughout or these absent towards apex and margins, margins scabrous throughout or in the distal 1/2–3/4, green or usually greenish-purple at the margins and towards the apex, 5-veined, apices acute and tapering into a short awn, sometimes slightly bilobate with awn emerging from between the minute lobes or very rarely muticous, awn 0.5–3 mm long, straight, scabrous; ***paleas*** (lowermost) 4.5–5.8 mm long, subequalling to usually equalling the lemma or slightly surpassing the lemma apex by up to 0.4 mm, herbaceous with scarious margins, slightly to deeply bidentate, keels scabrous in distal (1/4–)½–5/6 or rarely throughout, between keels smooth, moderately to densely granulose with clear bead-like raised silica cells appearing like ‘granules’, margins scabrous in distal ¼–1/2. ***Flowers*** proximally perfect with uppermost usually sterile; ***anthers*** 3 in number, 0.8−1.6(−1.8) mm long, linear, dull yellow; ***ovaries*** ca. 0.5−1 mm long, apex sparsely to densely pubescent; ***lodicules*** 0.7–0.85 mm long, bilobed with lobes ca. 2–4 mm long, both lobes +/- same size or lateral lobes to 0.2 mm shorter, glabrous, margins entire and smooth or sometimes fimbriate, acute. ***Caryopses*** ca. 2.6–3.5 mm long, ca. 1–1.6 mm shorter than lemma and palea, adhering to palea and lemma, narrowly elliptic to slightly narrow-obovate, deeply sulcate, hilum linear, 75–93% length of caryopsis, endosperm hard.

***Anatomy–Outline*** elliptical or obovate to carinate with angled arms, ca. 5 vascular bundles all positioned in the centre of the blade and at the same level, ca. 4 grooves, ca. 5 ribs; the central rib is located in the central area of the blade. ***Abaxial> surface*** with straight edges forming angles associated with the vascular bundles, ribs angular, composed of sclerenchyma block and found opposite all vascular bundles, smooth, macro-hairs absent, margins composed of sclerenchyma block. ***Adaxial surface*** markedly irregular, with rounded ribs situated opposite all vascular bundles, lacking sclerenchyma block, prickles present and densely covering the entire surface, sometimes more prevalent on the ribs, usually extending and appearing hispid (Fig. 2E, F).

##### Distribution and habitat.

Endemic to the high-elevation DMC of South Africa and Lesotho (Carbutt 2019). In South Africa, the species is known from the Eastern Cape and KwaZulu-Natal Provinces, with it also possibly occurring in the Free State Province, although no specimens have as yet been verified. *Festuca drakensbergensis* is a common constituent of both moderately grazed and little disturbed Afro-alpine vegetation (viz. Carbutt’s 2015 ‘austro-alpine region’), and less often in Afro-montane vegetation, of the DMC, ca. 2150–3270+ m alt. The species is found in grassland, wetland and short Afro-alpine shrubland dominated by species in the genera *Chrysocoma* L., *Erica* Tourn. ex L., *Eumorphia* DC. and *Helichrysum* Mill. These habitats correspondent with Mucina and Rutherford’s (2006) uKhahlamba Basalt Grassland (Gd 7), Lesotho Highland Basalt Grassland (Gd 8), Drakensberg Afro-alpine Heathland (Gd 10) and Lesotho Mires (AZf 5). *Festuca drakensbergensis* is rarely dominant and generally occurs in low abundance amongst the larger F. caprina var. macra or amongst other forbs or low shrubs. Of the 222 2 m × 2 m plots studied for all vascular plants across the Afro-alpine DMC (Sylvester et al. unpubl. data), *F. drakensbergensis* was encountered in usually low abundance (0.5–8[–70]% of overall plot cover) in 35 plots, highlighting its high frequency and ubiquity in these landscapes.

##### Preliminary conservation status.

The overall extent of occurrence of *F. drakensbergensis* is relatively large compared to many DMC endemics, perhaps 30% (or 13,000 km^2^ i.e. above 2150 m) of the total DMC area of ca. 40,000 km^2^. Given that it is a common species without any specific habitat niche, the total population is likely well above 10,000 mature individuals. However, given the tremendous pressure that the DMC is under from communal rangeland activities – especially in Lesotho (Global Mechanism of the UNCCD 2018, 2019) – it is possibly at medium- to long-term risk from land degradation through overgrazing. Initial observations suggest that the species does have resilience, being recorded in areas disturbed by grazing and burning as well as in areas of limited disturbance. There might, however, be competition from shrubland following overgrazing (e.g. *Chrysocoma ciliata* L., *Selago melliodora* Hilliard, *Eumorphia* spp. and *Helichrysum* spp.). Future projections of global climate change are also of concern for high-elevation species in southern Africa (Bentley et al. 2019). Accordingly, we propose the IUCN conservation status of Near Threatened (NT) until further population studies can be undertaken.

##### Etymology.

The species epithet refers to the Drakensberg Mountain Centre (DMC) of South Africa and Lesotho (Carbutt 2019), where this species forms a common component of the Afro-alpine vegetation.

##### Notes.

The character of extravaginal branching is not always easy to distinguish and certain specimens of *F. caprina* s.l. found growing in moss may have what appear to be rhizomes although these are, in fact, pseudostolons. However, F. caprina var. caprina and var. macra plants are usually much larger, with culms (28−)35−120+ cm tall, basal foliage (10−)30–80+ cm tall, generally (> ½) > ¾ to surpassing the length of the culms, with leaf-blades of tillers and basal culm (4–)12–66.5+ cm long, often > 26 cm long, basal sheaths entire, erect panicles with greenish or purplish spikelets on ascending branches, lower lemma often larger, (4.5−)5−7(−9) mm long, and anthers > 2 mm long (vs. culms (12.5–)20–46(–65) cm tall, basal foliage (2−)4−20(−27) cm tall, leaf-blades of tillers and basal culm (2–)5–15(–26) cm long, basal sheaths fibrous, drooping panicles with purplish spikelets on pendent branches, lower lemma 4.5–5.8, anthers 0.8−1.6(−1.8) mm long in *F. drakensbergensis*) (Table 1). Festuca caprina var. irrasa specimens can sometimes superficially resemble *F. drakensbergensis* by having shorter basal foliage reaching < ½ length of the culms, with smooth blades and fibrous basal sheaths (Table 1). However, in these cases, F. caprina var. irrasa can be distinguished by its intravaginally branched tillers which lack cataphylls, erect panicles with ascending branches, short-hispid or long-scabrous lemmas and paleas that often measure > 6 mm long, and anthers > 2 mm long (vs. extravaginally branched tillers with cataphylls present, drooping panicles with pendent branches, lemmas and paleas glabrous, scabrous, 4.5−5.8 mm long, and anthers < 1.8 mm long in *F. drakensbergensis*).

*Festuca exaristata* also bears extravaginally branched cataphyllous tillers or lateral-tending rhizomes, with plants forming short isolated tufts. The holotype of *F. exaristata* is very short, with basal foliage not reaching past 12 cm tall, and bears superficial resemblance to certain shorter specimens of *F. drakensbergensis*, for example, Sylvester et al. 3637. The protologue of *F. exaristata* mentions culms to 35 cm tall and leaf blades to 25 cm long, which must refer to the one paratype, du Toit 2713 (K), which has not been seen by us, showing that the species would also superficially match larger versions of *F. drakensbergensis*. However, *F. exaristata* differs by its entire, lustrous basal sheaths, blunt, rounded leaf-blade midribs, erect sub-spike-like panicles, smooth panicle branches, shorter lemmas 4−4.2 mm long which lack awns, glabrous ovary apex and anthers 1.5−1.8 mm long (vs. basal sheaths smooth or rarely retrorsely scabrous, fibrous, leaf blade midrib usually sharp, keel-like, sometimes blunt and rounded, panicles drooping, panicle branches lightly to densely scabrous, lowermost lemma (not including awn) 4.6−6 mm long, awn rarely absent, usually 0.5–3 mm long, ovary apex sparsely to densely hairy, anthers 0.8−1.6(−1.8) mm long in *F. drakensbergensis*). Although rarely some characters overlap between *F. drakensbergensis* and *F. exaristata*, the combination of characters found in *F. exaristata* is never found in specimens of *F. drakensbergensis*.

Some specimens (e.g. Sylvester et al. 3442) growing in wetlands with limited grazing were substantially larger than normal, with culms to 65 cm tall and inflorescences to 13 cm long.

##### Selected specimens examined.

**Lesotho.** Bokong Nature Reserve, ca. 350 m north from the information centre, 29.067203S, 28.421496E, 2972 m alt., Afro-alpine grassland dominated by Lachnagrostis barbuligera var. barbuligera with moderately controlled grazing and burning, 2 Mar 2020, S.P. Sylvester et al. 3687a (US); Bokong Nature Reserve, ca. 400 m north from the information centre, 29.065893S, 28.420137E, 2979 m alt., rocky Afro-alpine grassland dominated by Lachnagrostis barbuligera var. barbuligera with moderately-controlled grazing and burning, 2 Mar 2020, S.P. Sylvester et al. 3689 (PRE, US); Matebeng Pass, below highest summit close to the pass, 29.870708S, 28.976534E, 3094 m alt., “Lesotho Highland Basalt Grassland” with clear elements of “Drakensberg Afro-alpine Heathland” with *Erica* and *Helichrysum* shrubs dominating the landscape, 22 Feb 2020, S.P. Sylvester et al. 3578 (PRE, US); Menoaneng Pass, on road between Rafolatsane and Thaba-Tseka, 29.427423S, 28.951273E, 3040 m alt., Afro-alpine grassland, windy ridge, grazed down to low turf, 24 Feb 2020, S.P. Sylvester et al. 3595 (NU, PRE, US); Menoaneng Pass, on road between Rafolatsane and Thaba-Tseka, 29.427403S, 28.951124E, 3039 m alt., Afro-alpine grassland, windy ridge, grazed down to low turf, 24 Feb 2020, S.P. Sylvester et al. 3605 (PRE, US); Sani Pass area, close to the top of the Pass northwest of Sani Mountain Lodge, 29.521251S, 29.200602E, 3242 m alt., short Afro-alpine grassland, close to a pool of water, frequently to heavily grazed, 26 Feb 2020, S.P. Sylvester et al. 3636 (PRE, US); Sehlabathebe National Park, lower end of the Park on the border, 29.860061S, 29.095497E, 2719 m alt., damp Afro-alpine tussock grassland, soil damp, under dripping crag, heavily grazed, close to livestock paths, 19 Feb 2020, S.P. Sylvester et al. 3531 (NU, PRE, US). **South Africa.** Eastern Cape: Bastervoetpad Pass area, ca. 12 km east of Mountain Shadow Hotel on Barclay Pass, 31.176139S, 27.964197E, 2176 m alt., Afro-montane transitioning to Afro-alpine grassland, 14 Feb 2020, S.P. Sylvester et al. 3505 (NU, PRE, US); Eastern Cape: between Carlisleshoekspruit Pass and Tiffindell Ski Area, 30.677202S, 27.956643E, 2526 m alt., riparian wetland, 10 Feb 2020, S.P. Sylvester et al. 3442 (NU, PRE, US); Eastern Cape: Tiffindell Ski Area, Ben Macdhui summit, 30.647683S, 27.934042E, 2995 m alt., Afro-alpine grassland, 11 Feb 2020, S.P. Sylvester et al. 3459 (NU, PRE, US); KwaZulu-Natal: Drakensberg, top of Sani Pass, grassy slopes on bank of gully, steep east facing slope, between rocks in brown clayey soil, 9400 ft [2865 m alt.], 24 Mar 1975, P.C.V. du Toit 698 (PRE0240733); KwaZulu-Natal: Sentinel Trail, ca. 1.2 km from the chain ladders, 28.740834S, 28.886806E, 2867 m alt., Afro-montane grassland grading into Afro-alpine grassland, damp soil, infrequently grazed, 6 Mar 2020, S.P. Sylvester et al. 3714 (NU, PRE, US); KwaZulu-Natal: Sani Pass area, below southwest facing cliffs to the southeast of Sani Mountain Lodge, 29.585365S, 29.290839E, 2866 m alt., short Afro-alpine grassland, frequently to heavily grazed, 26 Feb 2020, S.P. Sylvester et al. 3637 (PRE, US); [KwaZulu-Natal?:] Probably from Mont-aux-Sources [Sentinel Peak?], E.A.C.L.E. Schelpe 1394A (PRE0024522).

### Taxonomic notes on other taxa in the *Festuca caprina* complex of southern Africa

#### Festuca caprina var. caprina

Taxon classificationPlantaePoalesPoaceae

Nees, Fl. Afr. Austral. Ill. 443. 1841. Festuca nubigena subsp. caprina (Nees) St.-Yves, Rev. Bretonne Bot. Pure Appl. 2: 79. 1927.

DD93F5FD-94F7-58D4-A874-531194B095E3

Fig. 3 , Table 1

##### Type.

South Africa. [Eastern Cape:] Table mountain, Queenstown Dev., Los-Tafelberg, 5000−6000 ft [1524−1829 m alt.], [1840], [flowering in December], D.F. Drége s.n. (lectotype, designated by Alexeev 1986: 1115: K (K000345257 [image!]; isolectotypes: K (K000345258 [image!]), LE (LE00009757 [image!]); syntypes: South Africa. Plantes du Cap, Los Tafelberg, 5000−6000 ft [1524−1829 m alt.], 7 Dec 1832, D.F. Drége 8.e.3920 (P (P00434763 [image!])); [South Africa] Afr. Austr. D.F. Drége s.n. (L (L1262355 [image!])); South Africa. Los Tafelberg, Dec 1826−1834, D.F. Drége s.n. (HAL (HAL0106999 [image!))).

= Festuca caprina var. curvula Nees, Fl. Afr. Austral. Ill. 1: 443. 1841. Type: South Africa. [Eastern Cape:] [Monte] Los Tafelberg, an steinigen Oetern, 5000−6000 ft [1524−1829 m alt.], [flowering in December], D.F. Drége s.n. (lectotype, **designated here**: S (S-G-6704 right-hand plant annotated with ‘b’ [image!]); syntype: South Africa. Plantes du Cap, D.F. Drége 8.e.3920? (P (P00434764 [image!])).

= Festuca costata var. longiseta Nees, Fl. Afr. Austral. Ill. 1: 447. 1841. Type: South Africa. [Eastern Cape:] Stockenstrom Division, Katberg, [4000−5000 ft; 1219−1524 m alt.], 1840, D.F. Drége s.n. (lectotype, designated by Alexeev 1986: 1115: K (K000345256 [image!]); isolectotype: K (K000345255 [image!])).

**Figure 3.**
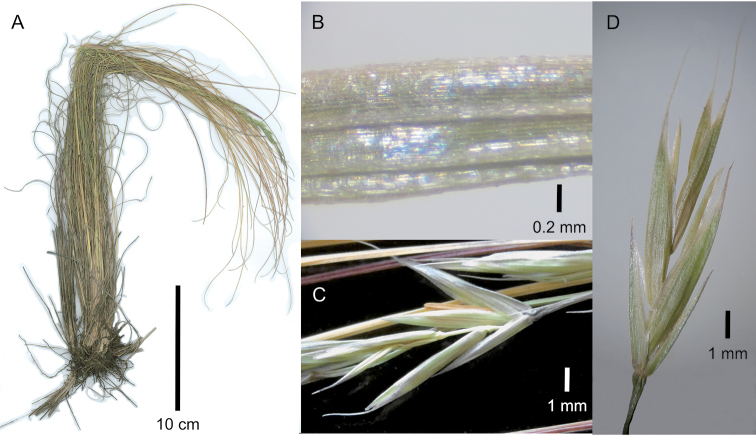
Festuca caprina var. caprina. **A** Whole plant **B** abaxial leaf blade surface of tiller **C, D** spikelet, lateral view. **A, B, D** of S.P. Sylvester et al. 3492 (US) **C** of S.P. Sylvester et al. 3492 (PRE).

##### Notes.

Alexeev (1986) separated *F. caprina* from *F. macra* (=F. caprina var. macra) based, in part, on the basal sheaths being fibrous. However, all type or original material of *F. caprina*, including the lectotype of F. caprina var. caprina designated by Alexeev (1986), had entire, often lustrous, basal sheaths apart from var. irrasa, which were obviously fibrous. The protologue mentions basal sheaths to be fibrous and, as such, Alexeev (1986) may have made an error in his choice of lectotype. Nevertheless, as only the type material of var. irrasa, which was designated by Stapf (1900), has fibrous basal sheaths, this also raises questions over the accuracy of the description in the protologue for var. caprina. If we treat F. caprina var. caprina based on the K lectotype and isolectotype designated by Alexeev (1986) then var. caprina should be considered as having entire basal sheaths that do not split into fibres. Oddly, the inflorescences of all var. caprina specimens studied had a distinct butter-like smell upon the opening of specimen press papers, which then quickly dissipated. This odour was barely to sometimes slightly susceptible in specimens of F. caprina var. macra or var. irrasa or *F. drakensbergensis*. It remains to be seen whether this character is diagnostic and what phytochemical compounds are involved.

Festuca caprina var. caprina is more common at lower elevations in the Drakensberg Mountain Centre (Carbutt 2019) and surrounding mountainous habitats of southern Africa and extends from southern Africa to Tanzania. The species appears to prefer more mesic Afro-alpine and Afro-montane grasslands and is outcompeted by F. caprina var. macra in the drier summit area of the high escarpment in the DMC. Of the 222 2 m × 2 m plots studied for all vascular plants across the Afro-alpine DMC (Sylvester et al. unpubl. data), *F. caprina var. caprina* was rarely encountered, being found in only 13 plots from the Eastern Cape and Free State. The species was usually encountered in lower elevation Afro-montane transitioning to Afro-alpine grasslands at ca. 2500−2700 m alt. or exceptionally at higher elevations to 2981 m alt. in damper shaded sites, highlighting its very low frequency and commonality in the high-elevation xeric Afro-alpine zone of the DMC.

Festuca caprina var. curvula is also herein lectotypified. In the protologue, Nees von Esenbeck (1841: 443) only cited a single Drége s.n. collection from monte Los-Tafelberg, 6000 ft (1829 m alt.), which is assumed to be the same type locality as var. caprina that was found in Los-Tafelberg of the Eastern Cape Province, near Queenstown. Nees von Esenbeck (1841: 443) labelled var. caprina and var. curvula ‘a’ and ‘b’, respectively, with the S-G-6704 right hand plant chosen as lectotype based on this matching the protologue information and being the only specimen sheet amongst the original material to be annotated with an ‘a’ and ‘b’ in Nees von Esenbeck’s cursive handwriting. The right-hand plant annotated with ‘b’ fitted the protologue description of var. curvula, with Nees differentiating the variety based on its shorter height, curved blades and subsecund panicle branches with few purplish spikelets. One specimen amongst the original material, D.F. Drége 8.e.3920? (P00434764), also had ‘Curvula’ written on the label but limited locality information aside from ‘Plantes du Cap’ and is here considered a syntype of var. curvula as it also fits the description given in the protologue. While the differentiating characters of F. caprina var. curvula are also found in *F. drakensbergensis*, we deduce that var. curvula is a slight variation from the norm in *F. caprina* as neither the type specimens designated herein, nor any of the other original material from the type locality, can be attributed to *F. drakensbergensis* based on their lacking extravaginal branching and cataphyllous shoots as well as having entire lustrous basal sheaths.

##### Selected specimens examined.

**South Africa.** Eastern Cape: Naudes Nek pass, near Rhodes, 30.764792S, 28.105164E, 2588 m alt., Afro-alpine tussock grassland, low rock outcrop, 13 Feb 2020, S.P. Sylvester et al. 3492 (NU, PRE, US); Free State: Witsieshoek, at beginning of Sentinel trail by parking lot, path-side, 28.733181S, 28.893296E, 2607 m alt., 5 Feb 2020, S.P. Sylvester et al. 3418 (US); Free State: Witsieshoek, Sentinel trail, along beginning of trail that leads to the chain ladders that take you up to Amphitheatre, path-side, 28.736207S, 28.894084E, 2693 m alt., 5 Feb 2020, S.P. Sylvester et al. 3416 (US); Free State: Golden Gate National Park, summit of Wodehouse Peak, 14 Jan 1975, R.P. Ellis 2383 (PRE0464133); KwaZulu-Natal: Amphitheatre, slopes near the Tugela waterfall, Afro-alpine grassland, 28.750810S, 28.888942E, 2981 m alt., 5 Feb 2020, S.P. Sylvester et al. 3409a (US); KwaZulu-Natal: Sentinel Trail, off the main trail at the top of an east facing gully ca. 1 km from the chain ladders, 28.743162S, 28.888205E, 2953 m alt., shaded Afro-montane grassland grading into Afro-alpine grassland, damp soil, rarely grazed, 6 Mar 2020, S.P. Sylvester et al. 3713 (NU, PRE, US).

#### Festuca caprina var. irrasa

Taxon classificationPlantaePoalesPoaceae

Stapf, Fl. Cap. 7: 720. 1900.

B82713AF-70DA-5358-80C9-FE653AF53E30

Fig. 4 , Table 1

##### Type.

South Africa. [Eastern Cape: Grahamstown], Howison Poort, Nov 1894, H.G. Flanagan s.n. (lectotype, designated by Alexeev 1986: 1115: K (K000345259 [image!]); syntype: South Africa. East Province of Cape Colony [Eastern Cape]: Amatole Mountains, Mar 1883, J. Buchanan 37 (K (K000345260 [image!])).

##### Notes.

Festuca caprina var. irrasa may indeed be distinct and warrant elevating to species level. It differs from the other intravaginally branched taxa in the complex (F. caprina var. caprina and F. caprina var. macra) by the obviously fibrous basal sheaths and usually short-hispid or long-scabrous (prickles hair-like) lemmas, paleas and rachillas. The character of lemma, palea and rachilla pubescence sometimes varies with hispid hairs sometimes only found at the apex of some lemmas in the inflorescence. The panicle branches and pedicels are also usually densely short-hispid or long-scabrous with hooks elongating to become almost hair-like, a character not seen in the other members of the *F. caprina* complex, although this character also appears to vary. The variation may be due to introgressive hybridisation or lateral gene transfer between taxa, which possibly occur frequently in grasses (Kellogg 2015; Hibdige et al. 2020; Tkach et al. 2020). This could be exemplified by how one specimen (Sylvester et al. 3547) that was collected close to both var. macra (Sylvester et al. 3538) and var. irrasa (Sylvester et al. 3542) had inflorescence characteristics of var. irrasa, but antrorsely scabrous abaxial leaf-blade surfaces like var. macra. Further work is needed to clarify the circumscription and taxonomic position of var. irrasa. *Festuca drakensbergensis*, described herein, also usually has fibrous basal sheaths and, although not as conspicuous as F. caprina var. irrasa, can be readily distinguished based on its extravaginal tiller branching, presence of rhizomes and smaller anther size, amongst other characters.

Festuca caprina var. irrasa is endemic to the DMC of southern Africa, being found in Lesotho and the South African Eastern Cape and KwaZulu-Natal Provinces and possibly the Free State Province (although no specimens have been verified by us). The species appears to be more common in the KwaZulu-Natal Province. During our ecological plot-based study across the Afro-alpine DMC (Sylvester et al. unpubl. data), *F. caprina var. irrasa* was only encountered as locally abundant ([0.5–]5–35% of 2 m × 2 m plot cover) populations in the damper southern sites of the DMC, i.e. Sehlabathebe National Park (Lesotho) and Barclays Pass (Eastern Cape, South Africa). The species was found in only 11 plots ranging from the lower elevation Afro-montane to Afro-alpine grassland transition at ca. 2250 m alt. to wet Afro-alpine tussock grasslands at ca. 2750 m alt.

Alexeev (1986: 1115) cites “(P. Linder in Fl. South Africa. manusc.): Cape Province, Grahamstown, Howisons Poort, no. 94, H.G. Flaganan (K!)” for lectotype selection. However, upon inspection of the K lectotype, the ‘94’ refers to the year of collection.

**Figure 4.**
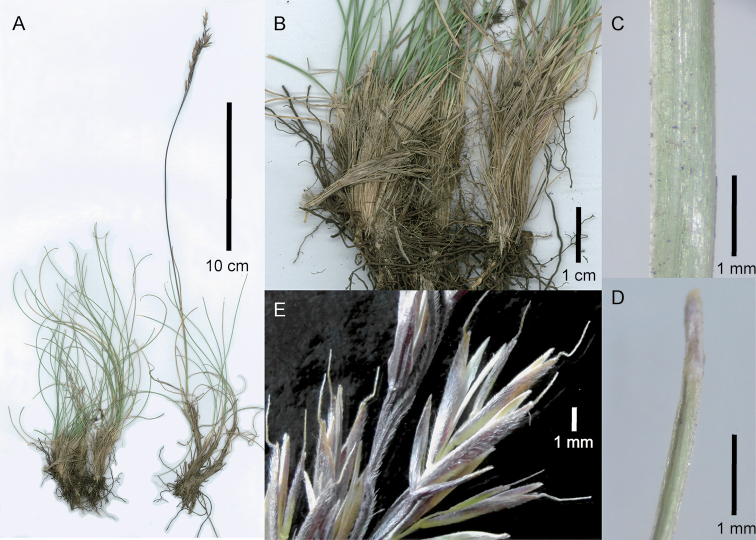
Festuca caprina var. irrasa. **A** Whole plant **B** close-up of basal sheaths showing fibres **C** abaxial leaf blade surface of tiller **D** leaf blade apex of tiller **E** close-up of inflorescence, showing spikelets, lateral view. **A, B, E** of S.P. Sylvester et al. 3512 (US) **C, D** of S.P. Sylvester et al. 3542 (US).

##### Selected specimens examined.

**Lesotho.** Sehlabathebe National Park, lower end of the Park on the border, 29.877593S, 29.086461E, 2606 m alt., wet Afro-alpine tussock grassland, soil damp, not grazed recently, 20 Feb 2020, S.P. Sylvester et al. 3542 (PRE, US); Sehlabathebe National Park, lower end of the Park on the border, 29.876061S, 29.086150E, 2645 m alt., gravelly slopes below basalt rock escarpment with grasses intermixed with forbs, soil damp, burned and grazed recently, 20 Feb 2020, S.P. Sylvester et al. 3547 (PRE, US). **South Africa.** Eastern Cape: Bastervoetpad Pass area, ca. 12 km east of Mountain Shadow Hotel on Barclay Pass, 31.172568S, 27.964747E, 2259 m alt., Afro-montane transitioning to Afro-alpine grassland under moderately-heavy grazing, 14 Feb 2020, S.P. Sylvester et al. 3512 (US); KwaZulu-Natal: Giants Castle, 10,000 ft [3048 m alt.], 8 Jan 1915, R.E. Symons 352 (PRE0023182); KwaZulu-Natal: Weenen county, top of Griffins Hill, ca. 5000 ft [1524 m alt.], sedgy banks of streamlet in highland sourveld, fairly frequent, 29 Oct 1944, J.P.H. Acocks 10740 (PRE0023178).

#### Festuca caprina var. macra

Taxon classificationPlantaePoalesPoaceae

Stapf, Fl. Cap. 7: 720. 1900. Festuca macra (Stapf) E.B. Alexeev, Bot. Zhurn. (Moscow & Leningrad) 71(8): 1116. 1986.

33F5A787-825A-5407-B4AB-EBE4EF42A792

Fig. 5 , Table 1

##### Type.

South Africa. [Kalahari Region: Orange Free State:] Wittebergen, near Harrismith, Comm. O. MacOwan, Feb 1877, Buchanan 262 (holotype: K (K000345247 [image!]); isotype: PRE! fragm. ex K).

##### Notes.

Alexeev (1986) raised var, *macra* to species level and differentiated it from *F. caprina* based on: a) sheaths of old leaves not falling apart into parallel thin threads (vs. falling apart (shredded) into parallel thin threads in *F. caprina*, although this is now considered erroneous; see comments under *F. caprina* above); b) leaf blades more or less glaucous (vs. green in *F. caprina*); c) abaxial leaf-blade surfaces scabrous (vs. smooth or scaberulous in *F. caprina*); d) adaxial leaf-blade surfaces shortly hairy (vs. scabrous or shortly hairy in *F. caprina*); e) lemmas 4.5−5 mm long (vs. 5−7[9] mm long in *F. caprina*); f) awns 1.5−3.5 mm long (vs. [0.5]1−4 m long in *F. caprina*); g) anthers 1.6−2.2 mm long (vs. [2−]2.5−4 mm long in *F. caprina*); h) spikelets straw-coloured-violet (vs. violet-green, rarely green in *F. caprina*). However, only the holotype of F. caprina var. macra was seen by Alexeev, as well as original material (from which a lectotype was selected) and a limited number of other specimens of F. caprina var. caprina at the K herbarium.

Upon study of numerous specimens that belong to F. caprina var. caprina and var. macra during extensive fieldwork in the DMC and herbarium study at PRE, it became apparent that the above-mentioned differentiating characters overlap. Both F. caprina var. caprina and F. caprina var. macra share most characteristics, such as intravaginal tillers forming dense, often large, tussocks, with entire, often lustrous, basal sheaths, narrow involute blades and similar inflorescence and spikelet morphology, with anthers usually > 2 mm long. The F. caprina var. macra holotype is on the shorter side with regards most inflorescence characters when compared with F. caprina var. caprina, with shorter spikelets, lemmas, awns and anthers according to the protologue. Nevertheless, most of these characters have also been found in specimens of F. caprina var. caprina, with variability in lengths of the spikelet parts possibly being related to ecological conditions, including seasonal variations in rainfall (C. Mashau, pers. comm.). The anther length of 1.6 mm, mentioned in the protologue for *F. macra* (Alexeev 1986: table 2), is shorter than any specimen of F. caprina var. macra studied by us, with it being plausible that the var. macra holotype could be somewhat intermediate between *F. drakensbergensis* and F. caprina var. macra in its broader sense, with similar plausible hybrids with a mixture of characters sometimes found in the DMC (see below). Indumentum of the adaxial leaf-blade surface was also found to vary between scabrous, long-scabrous with prickles becoming elongated and hair-like and shortly hispid in all the taxa of the *F. caprina* complex from southern Africa, with this character seen to have no diagnostic value.

Festuca caprina var. macra was not included in the treatment of southern African grasses by Fish and Moeaha (2015), who chose not to uphold any of the varieties of *F. caprina* stating that the species was too variable. Nevertheless, we consider F. caprina var. macra to be distinct from var. caprina based on the character of notably antrorsely scabrous abaxial leaf-blade surfaces that is not known outside of the DMC, with all other *F. caprina* specimens across their range being smooth or exceptionally scaberulous towards their apices. Specimens with notably scabrous leaf blades were also found to be geographically and ecologically distinct during fieldwork in the DMC, these being predominantly found in drier alpine areas of the DMC, while var. caprina was found in more mesic environments often at lower elevations in the montane belt.

Plants of the World Online (2020), Plantlist (2020), the World Checklist of Selected Plant Families (2020) and GrassBase (Clayton et al. 2006 onwards) currently accept *F. macra* as a distinct species. While we currently disagree with this assessment, more exhaustive research may result in var. macra being raised to subspecies level, with certain characters still needing to be assessed such as lemma micromorphology, which has been proven to be taxonomically informative in *Festuca* (Ortúñez and Cano-Ruiz 2013).

Festuca caprina var. macra is often dominant in less-disturbed Afro-alpine grasslands of the DMC (Carbutt 2019), being found in Lesotho and the Eastern Cape, Free State, KwaZulu-Natal Provinces of South Africa. Of the 222 2 m × 2 m plots studied throughout the DMC, 99 were occupied and often dominated ([0.5–]20–92% of overall plot cover) by F. caprina var. macra (Sylvester et al. unpubl. data), with a total of 42 collections of the species being made. It is more palatable than *Merxmuellera* Conert species and so is less common in grazed areas (Sylvester and Soreng, pers. obs.).

*Festuca obturbans* St.-Yves and its allies *F. gilbertiana* Alexeev ex S.M. Phillipps and *F. macrophylla* A. Rich., described from Afro-alpine vegetation of Kenya or Ethiopia, also bear superficial similarity to F. caprina var. macra in their intravaginally branched large tussocks with entire basal sheaths and fine, involute and usually scabrid leaf blades (Alexeev 1986; Phillips 1995a, b). These also share similar inflorescence characteristics with F. caprina var. macra, such as relatively-narrow panicles with spikelets loosely arranged on short ascending branches, and spikelets with similar glume, lemma and anther sizes (Alexeev 1986; Phillips 1995a, b). These can be differentiated by their leaf blade cross sections showing sclerenchyma girders bridging both sides of the vascular bundles or, at least, the larger ones (vs. sclerenchyma only present on the abaxial blade ribs in F. caprina var. macra). *Festuca gilbertiana* can be further differentiated by its smooth leaf blades, culms 30–35 cm tall, and sparse racemose inflorescence (vs. leaf blades scabrous, culms (28–)60–110(–120+) cm tall, inflorescence usually a large loosely-contracted panicle in F. caprina var. macra). *Festuca obturbans* can be further differentiated by having sheaths open to almost their base and ovary apices glabrous (vs. sheaths closed for ca. ½ their length, ovary apices sparsely to densely pubescent in F. caprina var. macra).

Two specimens found near the Tiffindell Ski Resort of the Eastern Cape, South Africa (Sylvester et al. 3428B) and Bokong Nature Reserve, Lesotho (Sylvester et al. 3687B), bore characteristics of F. caprina var. macra, which was collected alongside them (Sylvester et al. 3428A, 3687C), such as tussock-forming habit with intravaginal branching and entire basal sheaths not splitting into fibres. However, they differed by their smooth abaxial leaf blade surfaces, placing them closer to F. caprina var. caprina, unawned lemmas, which is unusual for both var. caprina and var. macra, and short spikelets with lowermost lemmas 4.5−5.8 mm long anthers measuring ca. 1.6−1.8 mm long, placing them closer to *F. drakensbergensis*. As *F. drakensbergensis* was also collected at the same localities (e.g. Sylvester et al. 3459, 3687B), it is plausible that these could be hybrids between F. caprina var. macra and *F. drakensbergensis*. More study, including further collections, is required to ascertain the identity of these specimens.

**Figure 5.**
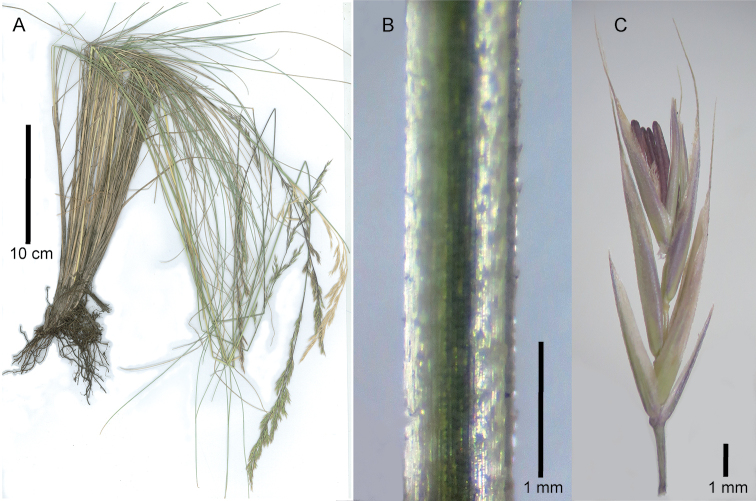
Festuca caprina var. macra. **A** Whole plant **B** abaxial leaf blade surface of tiller **C** spikelet, lateral view. Images of S.P. Sylvester et al. 3480 (US).

##### Selected specimens examined.

**Lesotho.** AfriSki area, in valley adjoining and northwest of the valley of the AfriSki resort, on the north side of the A1 highway, 28.808394S, 28.708658E, 3104 m alt., dry upper slopes above valley, 27 Feb 2020, S.P. Sylvester et al. 3652 (NU, PRE, US); AfriSki resort, in valley just west of the resort centre, 28.822906S, 28.724602E, 3046 m alt., relatively undisturbed damp Afro-alpine grassland, 28 Feb 2020, S.P. Sylvester et al. 3663 (PRE, US); Bokong Nature Reserve, ca. 350 m north from the information centre, 29.067203S, 28.421496E, 2972 m alt., Afro-alpine grassland dominated by Lachnagrostis barbuligera var. barbuligera with moderately-controlled grazing and burning, 2 Mar 2020, S.P. Sylvester et al. 3687b (US); S.P. Sylvester et al. 3687c (US); Matebeng Pass, below highest summit close to the pass, 29.870708S, 28.976534E, 3094 m alt., “Lesotho Highland Basalt Grassland” with clear elements of “Drakensberg Afro-alpine Heathland” with *Erica* and *Helichrysum* shrubs dominating the landscape, 22 Feb 2020, S.P. Sylvester et al. 3576 (US); Matebeng Pass, below highest summit close to the pass, 29.868524S, 28.976439E, 3125 m alt., Afro-alpine vegetation with Ericaceous shrubs dominating the landscape, heavy grazing, 22 Feb 2020, S.P. Sylvester et al. 3580 (US); Matebeng Pass, below highest summit close to the pass, 29.873765S, 28.976929E, 2947 m alt., Afro-alpine vegetation with Ericaceous shrubs dominating the landscape, heavy grazing, 22 Feb 2020, S.P. Sylvester et al. 3588 (PRE, US); Menoaneng Pass, on road between Rafolatsane and Thaba-Tseka, 29.427317S, 28.950617E, 3039 m alt., Afro-alpine grassland, windy ridge, grazed, 24 Feb 2020, S.P. Sylvester et al. 3601 (US); Sani Pass area, ca. 800 m east of Sani Mountain Lodge, 29.585198S, 29.292011E, 2896 m alt., short Afro-alpine grassland, frequently to heavily grazed, 25 Feb 2020, S.P. Sylvester et al. 3619 (PRE, US); S.P. Sylvester et al. 3620 (US); Sehlabathebe National Park, lower end of the Park on the border, 29.859882S, 29.095598E, 2779 m alt., wet Afro-alpine tussock grassland, soil damp, under dripping crag, heavily grazed, close to livestock paths, 19 Feb 2020, S.P. Sylvester et al. 3523 (NU, PRE, US); Sehlabathebe National Park, lower end of the Park on the border, 29.860180S, 29.095586E, 2733 m alt., wet Afro-alpine tussock grassland, soil damp, under dripping crag, heavily grazed, close to livestock paths, 19 Feb 2020, S.P. Sylvester et al. 3538 (PRE, US). **South Africa.** Eastern Cape: between Carlisleshoekspruit Pass and Tiffindell Ski Area, 30.6852485S, 27.963802E, 2565 m alt., Afro-alpine grassland, 10 Feb 2020, S.P. Sylvester et al. 3428a (PRE, US); Eastern Cape: between Carlisleshoekspruit Pass and Tiffindell Ski Area, 30.6852485S, 27.963802E, 2565 m alt., Afro-alpine grassland, 10 Feb 2020, S.P. Sylvester et al. 3428b (NU, PRE, US); Eastern Cape: Tiffindell Ski Area, 30.649239S, 27.928720E, 2845 m alt., Afro-alpine grassland, 10 Feb 2020, S.P. Sylvester et al. 3446 (NU, PRE, US); Eastern Cape: Tiffindell Ski Area, 30.676006S, 27.958567E, 2527 m alt., Afro-alpine tussock grassland, 12 Feb 2020, S.P. Sylvester et al. 3480 (NU, PRE, US); Eastern Cape: Tiffindell Ski Area, Ben Macdhui summit, 30.648172S, 27.935507E, 2998 m alt., Afro-alpine grassland, 11 Feb 2020, S.P. Sylvester et al. 3462b (NU, PRE, US); Eastern Cape: Tiffindell Ski Area, next to ski lift, 30.651034S, 27.925149E, 2778 m alt., Afro-alpine grassland, annually burnt, appears to be seeded with exotic species, 11 Feb 2020, S.P. Sylvester et al. 3463 (NU, PRE, US); KwaZulu-Natal: Amphitheatre, slopes near the Tugela waterfall, 28.754008S, 28.893853E, 2983 m alt., Afro-alpine grassland, 5 Feb 2020, S.P. Sylvester et al. 3403 (NU, PRE, US); KwaZulu-Natal: Amphitheatre, slopes near the Tugela waterfall, 28.753989S, 28.893563E, 2979 m alt., Afro-alpine grassland, 5 Feb 2020, S.P. Sylvester et al. 3406 (US); KwaZulu-Natal: Amphitheatre, slopes near the Tugela waterfall, 28.750810S, 28.888942E, 2981 m alt., Afro-alpine grassland, 5 Feb 2020, S.P. Sylvester et al. 3409b (US).

#### Festuca exaristata

Taxon classificationPlantaePoalesPoaceae

E.B. Alexeev, Bot. Zhurn. (Moscow & Leningrad) 71(8): 1116. 1986.

9F19FFA2-8871-5D06-8F2F-8B20EA3E6BA1

Fig. 6 , Table 1

##### Type.

[Lesotho] Basutoland. Above the Sani Pass, among stones, 9800 ft [2987 m alt.], 3 Feb 1959, M. McCallum Webster 483b (holotype: K (K000345250 [image!])).

##### Notes.

This species was not included in the treatments to southern African grasses (Gibbs Russell et al. 1990; Fish and Moeaha 2015), nor in the checklist to Lesotho grasses (Kobisi and Kose 2003), but is accepted in Plants of the World Online (2020), Plantlist (2020), the World Checklist of Selected Plant Families (2020), GrassBase (Clayton et al. 2006 onwards) and Tropicos (2020). It is known from just two collections; the type from Sani Pass, bordering Lesotho and the KwaZulu-Natal Province of South Africa, and a paratype from Letsing La Letsie of the Matatiele Province of Lesotho. Exploration by the authors in the Sani Pass area failed to discover further specimens although, at the time of visiting, the authors were not searching in particular for *F. exaristata* and did not cover all the habitats present. The holotype label states ‘Above the Sani Pass’ probably referring to the mountain slopes and ridge immediately above the Sani Pass, which were not explored by us. Our exploration largely focused on the valley bottom, which experienced very heavy grazing, with it being possible that the species may have been grazed out in these areas. As the species exhibits certain characters of both *F. caprina* s.l. and *F. drakensbergensis*, as well as other characters not found on any of these (e.g. glabrous ovaries, shorter unawned lemmas), there is also the possibility that the species is a hybrid which failed to survive into subsequent generations. However, the paratype, which was not seen by us, but was collected in 1977, 18 years after and ca. 130 km southwest of the type collection, raises doubt over this.

Alexeev (1986) distinguished this species from *F. macra* (= F. caprina var. macra) and *F. caprina* in part by: a) leaf blade mid-vein blunt and rounded; b) panicle branches smooth; c) lemmas 4−4.2 mm long; d) lemmas unawned; e) ovary apex glabrous; f) anthers 1.5−1.8 mm long. It can be further differentiated from F. caprina var. irrasa by the basal sheaths being entire, and from F. caprina var. macra by the leaf blade abaxial surfaces being smooth. Furthermore, although not mentioned by Alexeev (1986), the type material appears to have extravaginal branching, with cataphyllous laterally-tending shoots present, differentiating this from the intravaginally branched *F. caprina* s.l. The species does bear some resemblance to *F. drakensbergensis* (see notes under *F. drakensbergensis*).

**Figure 6.**
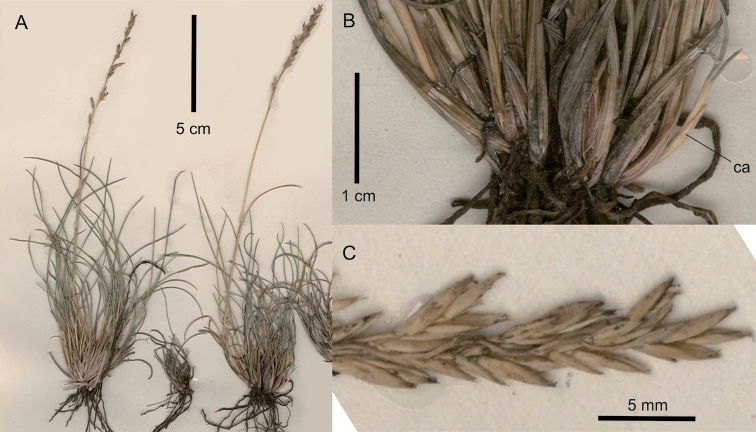
*Festuca exaristata*. **A** Whole plant **B** basal part of plant showing lustrous basal sheaths and extravaginally-branched tillers with cataphylls (ca) **C** close-up of inflorescence. Digitised images of holotype M. McCallum Webster 483b (K000345250), courtesy of JSTOR Global Plants (https://plants.jstor.org).

## Supplementary Material

XML Treatment for Festuca drakensbergensis

XML Treatment for Festuca caprina var. caprina

XML Treatment for Festuca caprina var. irrasa

XML Treatment for Festuca caprina var. macra

XML Treatment for Festuca exaristata
